# Losing and Regaining Sense of Control: A Cancer Patient’s Interactions With Healthcare Professionals

**DOI:** 10.1177/10497323251347896

**Published:** 2025-06-14

**Authors:** Satu Koskinen, Eeva Aromaa, Päivi Eriksson

**Affiliations:** 1 Free Researcher, Entrepreneur at Boardcoach, Joensuu, Finland; 2 Business School, 163043 University of Eastern Finland, Kuopio, Finland

**Keywords:** sense of agency, sense of control, cancer, collaborative autoethnography, illness narrative, healthcare professionals, Finland

## Abstract

By jointly telling the story of one of us, the authors, we highlight how supportive healthcare relationships can support a patient’s sense of agency and open pathways for restoring a sense of control in the midst of serious illness. Drawing on personal experiences of living through cancer as a patient or a caregiver in Finland led us to explore how a sense of control can be lost and gradually regained. Drawing on our shared reflections and experiences as collaborating autoethnographers, we analyzed Satu’s, the first author of this article, changing sense of agency and control, focusing on five key episodes over the 18 months following her diagnosis. As we revisited these episodes together, we identified how impersonal and one-sided communication within the healthcare system contributed to her loss of a sense of control. Over time, however, through more dialogic, respectful, and participatory interactions with healthcare professionals, she found ways to reclaim control and agency—not just in navigating illness but in shaping life beyond it. Our collaborative analysis offers new insights into the evolving dynamics of control, agency, and patient–professional interaction. We contribute to the literature on illness management by foregrounding relational, contextual, and socially constructed understandings of control. By illustrating the importance of patient–professional interactions, the study highlights the potential for rebuilding a sense of agency and control through supportive healthcare relationships.

## Introduction

Cancer patients pursue active involvement in managing their own care (Castro et al., 2016; Ocloo et al., 2021), including issues such as seeking to control their pain, dealing with intense emotions, filling information gaps, learning medical jargon, and overcoming illness-related stigmatization (Brennan & Moynihan, 2004). Drawing on the concepts of sense of control (Bandura, 1997; Pacherie, 2007; Skaff, 2007) and sense of agency (Deans, 2019; Moore, 2016), this study examines control in illness management from a patient’s perspective, focusing on cancer patients’ experiences (Greenhalgh, 2017; Gross, 2012; Horlick-Jones, 2011; Riessman, 2015; Wanat et al., 2016). Patient experiences of control have often been studied without any theory (e.g., Ogden et al., 2011). When theorized, various typologies of control (Volker & Wu, 2011) and the locus of control concept (Weda et al., 2023) have been used.

The concept of sense of control can be defined as people’s perception of their life as a structured, orderly, and predictable whole (Kay et al., 2008; Peluso & Pichierri, 2021; Skinner, 1996). It is closely connected to the concept of sense of agency (Chambon & Haggard, 2012; Gallagher, 2007; Pacherie, 2007), which refers to individuals’ retrospective perceptions of themselves as active agents who take purposeful action to steer their lives, particularly after unexpected events, such as illnesses (Acke & Meganck, 2022; Deans, 2019).

Prior empirical research has explored sense of control from a psychological perspective, using quantitative (see Mystakidou et al., 2015; Precht et al., 2023) and qualitative (see Romo et al., 2017; Seibel et al., 2023) research designs that focus on patients’ perception of independence and autonomy. However, previous research drawing on the social constructionist perspective has studied cancer patients as reflexive actors who navigate challenges by reconstructing their sense of self (Avery et al., 2023; Rashidi et al., 2021), coherence (Vestøl et al., 2021), and self-efficacy (Karademas et al., 2023). Our study contributes to the latter stream of qualitative research on patient experiences.

In this social science–oriented autoethnographic study (Hughes et al., 2012), we focus on the nuanced contextual and relational meanings that patients attach to their health condition, cancer care, and personal and work life. These are activated, for example, when patients engage in specific activities in their care and when they reflect on their own actions in relation to those of others (Acke & Meganck, 2022; Bamberg, 2011). Furthermore, we explore the evolvement of a patient’s sense of agency and control over time.

Autoethnography offers a valuable methodology for examining patient experiences in the context of health and illness (Adams et al., 2021). Previous autoethnographies have studied cancer patients’ fear of relapse (Horlick-Jones, 2011), objectification during medical procedures (Gross, 2012), identity reconstruction (Riessman, 2015), existential challenges related to chemotherapy (Greenhalgh, 2017), professional–personal boundaries in academia in the case of a student’s cancer diagnosis (Smith-Tran & Hang, 2022), experience of childhood cancer and the consequences of absence from school (Andersen, 2024), the role of culture among ethnic minorities navigating cancer trauma and treatment (Yu, 2024), and shifts in cancer patients’ sense of agency and the meaning of cancer (Aromaa et al., 2025). Although healthcare professionals significantly influence how patients cope with their illnesses (Dwarswaard et al., 2016; McCorkle et al., 2011), previous autoethnographies have often overlooked the role of these professionals as co-constructors of patients’ sense of agency and control.

Patients’ experiences of their interactions with healthcare professionals in the context of illness management lie at the core of the collaborative autoethnography (CAE; Chang, 2016, 2021; Chang et al., 2016) we present in this article. In our CAE, which was undertaken by three authors, we took a social constructionist perspective (Romo et al., 2017) to explore how real-time experiences of control and agency are retrospectively reconstructed. Our main aim was to provide new insights into how the collective reconstruction of past experiences can strengthen patients’ resilience and improve their health (Robinson & Lachman, 2017).

## Previous Research on Sense of Control

There is a considerable amount of literature on cancer patients’ control perceptions. This literature has focused on treatment-related decision making (Schuler et al., 2017), pain management (Vallerand et al., 2007), information sharing (Beaver & Booth, 2007), self-care (Kidd et al., 2009), personal time and family relationships (Volker & Wu, 2011), and improving quality of life (Henselmans et al., 2010).

Patients’ sense of control, as a theoretical concept, refers to people’s perception of their life as a structured, orderly, and predictable whole (Kay et al., 2008; Peluso & Pichierri, 2021; Skinner, 1996). Empirical research on patients’ sense of control has mostly been conducted within the field of psychology. These studies have addressed the socio-psychological and socio-emotional aspects of care, treatment, and living with cancer (e.g., Precht et al., 2023). In these studies, sense of control is studied as a static phenomenon in relation to the cause and course of illness (Mystakidou et al., 2015), the cure and nursing process (Henselmans et al., 2010), information seeking (Dickerson et al., 2011), the reading of medical records (Kayastha et al., 2018), coping strategies (Gibbons & Groarke, 2018), and physical capacity (Monforte-Royo et al., 2018).

These psychological studies are based on a binary distinction between either the patient as a master or an undergoer in relation to prevailing conditions. Agency remains undertheorized in those studies in which mastery reflects a view of the self that has control over one’s life. By contrast, fatalism refers to beliefs indicating that life is beyond our control (Caplan & Schooler, 2003). Fatalistic beliefs have been offered as an explanation for the lack of action and giving up when faced with severe health problems (Skaff, 2007).

## Sense of Control and Agency as Socially Constructed, Contextual, and Relational Phenomena

To understand new and confusing situations in which patients experience loss of control, they engage in sensemaking and attempt to create order out of the confusion (Weick et al., 2010). When facing devastating news about health and illness, patients make sense of what is happening by discussing with others, such as healthcare professionals and other patients. This helps maintain a sense of control over one’s life and the surrounding environment (Peluso & Pichierri, 2021). Thus, the process of losing, gaining, and maintaining one’s sense of control is shaped by interactions with other people. Sense of control is also related to patients’ attempts to avoid uncertainty through their own actions and those of others (Wegner & Wheatley, 1999). In the process of seeking control, the self is viewed as competent and efficacious, while others are viewed as collaborative and responsive (Bandura, 1997).

In ideal situations, patients not only succeed in making sense of their illness but can also steer the course of their lives in a preferred direction, toward a new situation (Skinner, 1996). Therefore, gaining and maintaining one’s sense of control requires performing actions and making choices that produce intended rather than unintended consequences (Palmer, 2007). Patients’ sense of control also depends on the quality of their interactions with others and how that may change outcomes and the situation itself in relation to their preferences and demands (Skinner, 1996). Therefore, losing, gaining, and maintaining a sense of control is a dynamic process (Fung et al., 1999).

Prior research has often dealt with sense of agency and sense of control as closely connected (Chambon & Haggard, 2012; Gallagher, 2007; Pacherie, 2007), such that the self is viewed as an agentic actor seeking control over one’s life (Haggard & Chambon, 2012). Grounded in agential phenomenology, feminist studies, and anthropology, agency has come to be understood as an experience of empowerment (Campbell & Maynell, 2010). It varies depending on our capacity to explore alternative courses of action, achieve intended consequences, and perceive our actions as meaningful, with the potential to produce intended effects (Deans, 2019; Moore, 2016). Since sense of control supports patients’ feelings of competence, self-confidence, and empowerment (Inesi et al., 2011), sense of agency and control can be understood as co-constitutive, that is, as shaping each other (Stapleton, 2018).

Conceptualizing sense of agency and control as contextual and relational phenomena entails that our interpretations of the self as agentic and capable of control are socially constructed in relation to specific situations, the surrounding environment, and other people (Stapleton, 2018; see also Acke & Meganck, 2022). While the context is related to how we perceive our capacity to take action in a given situation and respective circumstances (Gallagher, 2007), relationality refers to how we interpret our actions in relation to other people, such as other patients and healthcare professionals, to organize our experiences and attribute meaning to various actions (Seilonen & Wahlström, 2016). Relationality refers to an understanding of patients as intentional and reflexive in relation to other people (Bamberg, 2011), who are understood to be responsive and collaborative (Bandura, 1997).

Pacherie (2007) and Nahmias (2007) have emphasized the complex interplay between sense of agency and control. Examining these concepts as socially constructed, contextual, and relational phenomena provides a valuable framework for understanding how patients make sense of their illness journey. Pacherie (2007) found that patients’ efforts to gain control may not align with their goals and preferences. Even if the outcomes of a patient’s actions fall short of their intended goals, they might still experience an enhanced sense of control if they believe that they did everything within their power to influence the situation. Thus, a patient’s sense of control can be strengthened through the experience of exerting maximum effort, regardless of the actual result. Conversely, patients may experience that their sense of agency and control stems from relatively easy or effortless actions. According to Nahmias (2007), the absence of struggle may contribute to feelings of competence and mastery over the situation, which reinforces one’s sense of agency. Thus, both experienced effort and outcomes significantly shape how sense of agency and control are constructed over time.

## Methodology

This study is based on the principles of CAE, which emphasizes systematic reflection on personal experience in collaboration with others (Chang, 2016, 2021; Chang et al., 2016). Following the criteria articulated by Hughes et al. (2012), we explain in this section how our work was connected to the four key criteria of autoethnographic research: formulating social scientific problems, engaging in discussions on methodology, offering multiple perspectives, and providing credible analysis and interpretation.

In September 2021, Satu and Eeva, the first and second authors of this article, were diagnosed with bowel cancer and underwent treatment at different hospitals in Finland over the following months. Both have since returned to work and are enjoying life as much as they did before their diagnoses. In October 2021, as they navigated their cancer experiences, Satu and Eeva engaged in deep conversations, which Päivi, the third author, joined in 2022. Päivi’s involvement was related to losing her mother to stomach cancer in 2020, prior to which she was her mother’s primary caregiver for two years.

Drawing on our personal experiences as two cancer patients and one caregiver for a cancer patient, we undertook a CAE to focus our analytical lens on the problem of control in illness management, emphasizing its contextual and relational aspects (Chang, 2016; Crawley, 2012; Hernandez et al., 2017). Through ongoing discussion and reflection, we identified the loss and regaining of agency and control as central to Satu’s experience. This insight formed the core of our research problem and guided the analytical direction of the study.

CAE created a space for deep listening and trusting relationships, enabling a rich examination of illness management experiences (Ellis, 1999; Watfern et al., 2023). Our collaboration brought together multiple perspectives: two patients and one caregiver. In addition to our illness experiences, all three of us have professional expertise in understanding the broader patient perspective. Satu, who holds a PhD in leadership and serves as a non-executive board director for several companies, is also an entrepreneur and trained as a peer support person for other cancer patients. Eeva, who has a master’s degree in health sciences and a PhD in management, has been trained as a cancer expert by experience. Päivi, a professor of management at the university, leads a research group in which Eeva works full time and recently launched a major, externally funded research project on patient and public involvement in healthcare research.

Data generation began between October 2021 and April 2022, during which time Satu and Eeva met online every three weeks to share their experiences. The discussions drew on several data sources: Satu’s personal diary, Eeva’s discussion notes, and their WhatsApp and email correspondence. All of these sources are considered valid in autoethnographic research (Lee, 2019; Winkler, 2018). Having the key informant (Satu) as a co-author alongside two full-time academics allowed us to collaboratively uncover new insights. Eeva and Päivi acted as reflective “mirrors” (Bissett et al., 2018), helping to articulate implicit experiences and generate layered interpretations. This collaboration ensured that our analysis was grounded in real-life complexities and open to multiple vantage points.

Having several methodological discussions, we decided to use retrospective direct interpretation (Eriksson & Kovalainen, 2015; Stake, 2005), a qualitative strategy suited for interpreting past experiences in a holistic and intuitive manner without reducing the data into coded categories or themes. This method, often used in phenomenologically oriented qualitative research (Tuffour, 2017), allowed us to stay close to Satu’s lived experiences and avoid being overly driven by theoretical preconceptions. We also used techniques such as mind mapping and memo writing to document evolving reflections, interpretations, and analytical questions—providing an audit trail from early insights to the final analysis. This flexible approach enabled a responsive and iterative process of knowledge generation, grounded in shared meaning-making and attentive to emergent themes (Eriksson & Kovalainen, 2015).

In the beginning of the analysis, Satu wrote the first draft of her illness narrative. This was based on data collected in conversations and written exchanges with Eeva. The draft was structured into episodes and shared with Eeva and Päivi. During our analytical discussions, we noticed that the key element in Satu’s story was her experience of losing agency and control following her diagnosis, and her gradual process of regaining both. Eeva and Satu jointly drafted the first version of the theoretical framework on the sense of control, which we jointly refined to also include the sense of agency, and to better guide the analysis. Päivi further suggested that Satu’s interactions with healthcare professionals were central to the co-construction of agency and control. This insight deepened our analytical focus on relational and contextual aspects of care (Cornwell & Shaw, 2024). Satu then revised the narrative episodes to highlight these relational dynamics more explicitly.

We selected the episodes presented in this paper based on their relevance to our research questions. Credibility was enhanced through researcher triangulation (Stake, 1995), where differing interpretations were not suppressed but used to enrich the analysis. We adopted a rotating model of collaboration, shifting roles as a leading editor, creative analyst, and critical reader. These roles allowed us to challenge presumptions, address biases, and offer constructive feedback to one another. This iterative and reflexive process was crucial in refining the manuscript and sharpening the analytical focus. The process added rigor and coherence to the study, allowing for credible interpretation and sustained analytical depth (Karalis Noel et al., 2023). In line with Ellis and Rawicki (2013), our collaborative approach transformed individual lived experiences into meaningful academic knowledge.

## Ethical Considerations

Autoethnographies involve representing the voices of the self and others, which places the responsibility of safeguarding the rights of all those involved (Chang, 2016). This article is part of the project “Cancer Experiences During Unsettled Times: Collaborative Autoethnography,” which received approval from the Committee on Research Ethics of the University of Eastern Finland (approval statement 33/2022, dated September 26, 2022). While preparing the ethics approval application, we familiarized ourselves with the specific guidelines offered by the Finnish National Board on Research Integrity (Kohonen et al., 2019). In our self-assessment report for the ethics committee, we thoroughly examined the ethical challenges associated with protecting our own privacy and that of other individuals mentioned in the publication due to their actions, opinions, or understandings (Denshire, 2014). The procedures we followed, as well as their justification, are detailed below.

As autoethnographers often do, we will publish this study with our own names. This removes the possibility of safeguarding the anonymity of the authors mentioned in the study (Tolich, 2014). However, this creates a need to consider how to protect ourselves. According to prior research, publishing personal material without anonymity can expose authors to risks such as stigmatization, negative judgments from colleagues, and potential career repercussions (Lapadat, 2017). We considered these potential consequences but did not perceive them as harmful to us. We are all used to sharing experiences in various contexts. Päivi and Eeva contribute their perspectives through participatory health research with patients. Satu offers her insights as a trained volunteer providing peer support for other cancer patients. Eeva shares her personal experiences as a trained expert by experience on cancer in professional and patient-focused settings. Bowel cancer is an increasingly common disease in Western countries (Sharma et al., 2022) and is frequently discussed in Finnish society and media. However, to ensure our joint respect for each other’s autonomy and dignity, we agreed that each author could withdraw from the study at any time. In such a case, any unpublished, collaboratively produced material would not be used by the other authors.

We do not mention any family members or other close connections, such as relatives, friends, or colleagues, in this paper. Moreover, to protect their privacy and interests, we refrain from disclosing any specific health issues, personal circumstances, or experiences that could be sensitive, newly discovered, or potentially distressing to the authors’ close ones. This approach ensures that their confidentiality and well-being are fully respected.

We jointly discussed another ethical challenge in autoethnographic research. Some people are unaware that they are mentioned in the paper, namely, healthcare professionals. Asking for consent retrospectively would have placed them in an unfair position, making them feel obligated to consent (Tolich, 2010). To safeguard their privacy, we removed all personally identifiable information from the paper (Lapadat, 2017). In Finland, one can choose the hospital where one is to be treated; however, we do not name the hospital where Satu was treated or the healthcare professionals’ positions in the hospital hierarchy. Moreover, the specific fields of medicine in which the healthcare professionals mentioned are working are named only when necessary for the analysis.

## Findings: Patients’ Evolving Sense of Agency and Control After Cancer Diagnosis

Before outlining the analysis of cancer patients’ sense of control, we introduce Satu and discuss her experiences in receiving her cancer diagnosis. Satu was a 55-year-old female professional who worked as a solo entrepreneur and a board member or chair in several other companies. She was used to being in charge and was able to influence her schedules and life routines. Satu has been in good health for most of her life. Cancer was not very familiar to her before the diagnosis, and most of her understanding of it came from the media. At the time of the diagnosis in September 2021, the COVID-19 pandemic was still ongoing in Finland; however, in most aspects, Finnish society operated relatively normally.

Satu’s cancerous tumor was detected in a rectosigmoidoscopic examination performed at a private clinic. The doctor commented that the finding was most likely a malignant one, which would be confirmed after the laboratory examination. Satu was just told to wait and that she would soon be contacted by the local public hospital. Without any support or help, Satu left the clinic, confused and shocked. In addition to being extremely worried about her health and coping, Satu felt a total loss of control over all aspects of her life. What would happen to her? How bad would it be? Would she survive? How would she cope with the treatments? What would this do to her family and other close relationships? Could she continue working? When and how would she get any answers to these questions? Was there anything left that would be in her control from now on?

### Episode 1: A Passive Recipient of No-Reply Text Messages

The first episode in Satu’s story illustrates how one-sided communication, inviting her with no-reply text messages for diagnostic procedures, contributed to a loss of sense of agency and control over the medical information, diagnostic process, and work routines as a board member and entrepreneur:

As advised by the doctor who found the tumour, I waited to be contacted by the hospital. I was extremely worried and afraid. Finally, a week later, I received a no-reply text message. It invited me to blood tests, MRI and CT. The acronyms were strange to me. With the help of Google, I discovered that they asked me to go to magnetic resonance imaging and computer tomography examinations. One day later, another no-reply text message invited me to a rectosigmoidoscopic examination. I was not sure if the forthcoming procedures were examinations or cancer treatments. However, I felt satisfied that, clearly, I had now been taken to the hospital and that, hopefully, I would soon get more information on how serious my cancer was. I also got a phone call from a nurse at the hospital, and we discussed how the diagnostic and treatment processes could proceed in this type of cancer.

When I went to the MRI and CT scans, the nurse said I would be informed of the results later. However, four days later, I received a text message. It invited me to an “EUS examination” next week. I had no idea what it was. I rapidly Googled it and got very worried because I thought that going to an Endoscopic Ultrasound examination, as became clear to me from Google, meant that the scans had also found cancer elsewhere in my body, not just in my bowel.

A couple of weeks later, a text message instructed me to go to blood tests next week. I soon received yet another message, urging me to get blood tests done the next day. Again, I took this sudden hurry as a signal that something very bad had been found in the examinations and that my treatment had been classified as urgent. This made me extremely worried. I felt fear, uncertainty and despair. Yet again, the no-reply text messages signalled to me that I was supposed to put my previous life totally on hold to be available for examinations at any time. I skipped a scheduled board seminar in another city and didn’t dare schedule any new training dates with my company’s customers. I was extremely worried about whether I could provide the training I had promised my customers.

My situation felt so hopeless. I felt that I could do nothing to make it better. I felt that I didn’t have the knowledge to help myself. I thought that, with so serious and unpredictable an illness as cancer, the situation was out of my hands. I thought my attitude would not make any difference.

This episode illustrates how Satu experienced a total lack of control as a passive recipient of impersonal no-reply text messages from the hospital. Such communication offered her no room for dialogue or clarification, which left her feeling confused and powerless. She struggled to make sense of the acronyms in the text messages and relied on Google to understand unfamiliar medical terms. This added to a lack of agency, as she felt detached from the medical processes unfolding around her, unable to directly influence or understand what was happening. The urgency implied by sudden changes in scheduling her examinations increased her anxiety and feelings of helplessness. Each text message became a new source of worry, creating an impression that her condition was deteriorating without any explanation why. The lack of human interaction intensified her feelings of vulnerability, as she felt she was merely going through procedures dictated by the hospital without being able to grasp her situation. Fear and uncertainty dominated, as she had no control over the treatment trajectory.

From a socio-cultural perspective, this episode highlights the bureaucratic and technology-driven nature of modern healthcare systems (Cruz, 2023), in which efficiency and automation precede human care. The reliance on no-reply text messages to convey crucial medical information indicates a shift toward digital healthcare (Menvielle et al., 2017). Patients are expected to navigate with minimal direct interaction with medical staff, despite this digitalization often being justified as improving communication among different actors, such as healthcare professionals and patients (Vaagan et al., 2021). Even though technology-driven communication, such as sending patients invitations to examinations via text messages, may sound efficient from the hospital’s standpoint, this episode demonstrates how patients are left feeling alienated and uncertain when dealing with something as serious as cancer. The impersonal nature of this communication implies a system in which the patient is reduced to a procedural role, awaiting instructions without the possibility of engaging in dialogue. This conflicts with efforts to implement patient-centered care that respects individual preferences and patient diversity. This is a widely recognized guiding principle in nursing practice in Finland, among other countries (Vaagan et al., 2021). The cultural shift toward self-sufficiency in understanding medical procedures implies that patients must take more responsibility for their own care (From, 2015); however, such dynamics leave them struggling alone. Thus, we conclude that life outside of illness becomes secondary in a system in which patients are expected to be available anytime without any clue about why.

Relationally, we interpret this episode as describing a situation in which technology replaces human interaction. The no-reply text messages create a stark divide between the rights of the patient and the healthcare provider, amplifying experiences of isolation. Without a bond with care providers, the patient becomes a passive recipient of instructions rather than an active participant in their treatment (see Mishra et al., 2016). This lack of human relationality compounds feelings of helplessness, as there is no opportunity for dialogue or reassurance. A phone call with a nurse provided a point of human contact, but it was insufficient to reduce the overwhelming uncertainty.

The episode demonstrates how interactions between patients and healthcare professionals can be highly limited and unidirectional. They deliver information without providing space for questions, clarification, or emotional support, leaving the patient grappling with the implications of each message in isolation. The one phone call from the nurse offered some relief but did not help Satu understand the forthcoming messages and changing instructions. The lack of personalized communication created a feeling of being unseen while struggling through diagnostic tests and treatments without anyone acknowledging her emotional and psychological needs. The first episode highlights the importance of direct personal communication in healthcare, especially for patients undergoing life-altering diagnoses such as cancer (see Prip et al., 2018).

### Episode 2: Bad News Is Better Than No News

The second episode illustrates a shift in Satu’s story toward regaining a sense of agency and control over her cancer care, health condition, and personal and work life. This shift was supported by a nurse at the hospital cancer unit who told Satu about the possibility of modifying the timing of her daily chemoradiation therapy.

Feeling worried about my situation and what would happen next, I called the hospital’s cancer unit. The nurse told me that my chemoradiation therapy would start in two weeks and would last five weeks. The nurse patiently answered all my questions about the therapy. She said that there is a lot of variety in patients’ conditions during the therapy and that some patients are able and willing to work during the treatment period. She promised that I would be able to influence the timing of my daily therapy. She said that if I felt good enough, I could drive with my own car to the hospital. I remember that all this information raised strong hope in me in terms of restoring at least some normal elements in my life despite the illness.

I had previously been briefly informed that there would likely be two options for my treatment before the surgery. In the better case, one week of radiation therapy. In the more difficult case, five weeks of chemoradiation therapy, which is a combination of chemotherapy and radiation therapy. I understood during the call that this was obviously the more difficult case. Despite this negative information, I felt much better after the call. I had a much better understanding of what would happen next. I could also start to make some plans in my work and personal life, even if the effects of the treatment on my condition could not be known beforehand.

The second episode demonstrates how Satu regained her sense of agency and control through a phone call with the nurse, as she received concise information about the treatment plan and the ability to modify the timing of her daily therapy sessions. The nurse’s detailed responses helped her feel empowered to make decisions, allowing her to begin planning her personal and work life despite the uncertainty of the treatment’s effects. This resonates with previous literature on how patients’ ability to ask questions is a critical element in managing uncertainty in cancer care (McCormack et al., 2011) and how female and more educated patients appreciate detailed information (Fujimori & Uchitomi, 2009). The episode also demonstrates that, despite being informed of the need for the longer and more severe treatment option, Satu felt better after the call because of regaining a sense of agency and control over her personal and work life.

According to our discussion and collective analysis, this episode reflects a more patient-oriented approach to care in which the healthcare professional takes time to answer the patient’s questions and provides flexibility in the treatment schedule, which is culturally valued in modern healthcare systems (Castro et al., 2016). The ability to balance medical care with personal life, such as driving oneself to appointments and maintaining some work activity, illustrates a cultural emphasis on maintaining normalcy and personal autonomy during treatment (Iannarino et al., 2017).

The patient’s sense of control is clearly shaped by a supportive and informative relationship with the nurse. By offering flexibility in scheduling, the nurse enables her to regain agency, showing how control and agency are relationally constructed through communication and mutual understanding between the healthcare provider and the patient. The positive interaction between the patient and the nurse demonstrates the value of clear, empathetic communication in reducing anxiety (see Lelorain et al., 2012) and improving the patient’s understanding of her situation. The nurse’s willingness to provide detailed answers and to allow the patient to influence some aspects of the treatment enhances her comfort and preparedness for upcoming therapy.

### Episode 3: Leave It to Us to Cure You!

The third episode illustrates how Satu regained sense of agency and control in her treatment through the support provided by an oncologist and a nurse.

There was a lot of expectation in the air when I finally had an appointment with an oncologist five weeks after the tumour had been found. I was happy to find that the oncologist looked warmly straight into my eyes, noticed me as a person and had clearly familiarized herself with my case. I felt great relief to hear her say, “Leave it to us to cure you!” For me, this included two important messages: Firstly, they would be able to cure me. Secondly, they would take care of it. On the other hand, I thought that I did not really have any other option. The oncologist advised me how I could take care of myself during chemoradiation therapy. I felt relieved to hear that my duties would include very mundane things: eating and drinking enough, taking walks, skin moisturizing and medication. I believed I would be able to take care of all this.

Next, a nurse explained in detail the practicalities related to my forthcoming chemoradiation therapy. She also gave me instructions and maps in print to help me find my way to the right places. She had packed a tiny bag of cytostatic drugs for me and handwritten my name and dosing instructions on the label. This was to help me get started with the treatment as planned. Everything was very organized, clear and made as easy for me as possible.

After leaving the hospital, I carefully took care of everything mentioned by the oncologist and the nurse. At the pharmacy, I bought all kinds of body lotions, toothpaste for dry mouth and anti-nausea medicines even though I had no idea whether I would need any of them. Although the oncologist had said there was no need to stay isolated due to the COVID-19 pandemic, I was worried about the possibility that I would infect myself and other patients in the cancer unit. I avoided social events as much as possible, always kept a safe distance and stopped playing padel even though it let me forget the cancer for a while. In addition to the COVID-19 pandemic, another threat emerged later. I was afraid that the caregivers’ strike would postpone my second surgery if I got infected and the strike would start.

The third episode illustrates how the patient experienced both relief and a sense of regained agency after meeting the oncologist. From the perspective of cancer patients, clear roles and responsibilities in the implementation of cancer treatments are linked to the quality of care (Hess & Pohl, 2013). While the oncologist assumed responsibility for the overall treatment, the patient was given control over simple self-care tasks, such as eating, walking, and moisturizing. She felt empowered by having clear instructions and organization, which enhanced her sense of agency in managing aspects of her health. The patient’s anxiety about postponements due to the pandemic and the threat of a strike highlight the vulnerability of agency to system-wide disruptions.

The oncologist’s warm interaction, focus on personalized care, and detailed guidance tell us about the cultural value placed on patient-centered care, which is designed to address both the emotional and practical needs of patients (Mohammed et al., 2016). This episode also reflects the broader socio-political dynamics in healthcare. The patient’s preoccupation with COVID-19 precautions and concerns about a healthcare strike show how both impact the patient’s ability to maintain a feeling of normalcy and security during treatment.

Previous research has demonstrated how cancer patients’ uncertainty due to a lack of information and support from healthcare professionals is connected to a lack of self-care behavior to cope with the effects of the disease, which leads to a decreased sense of self-efficacy (Zhang et al., 2015). The third episode illustrates how the oncologist’s reassuring message and the nurse’s detailed preparation of medications and instructions made the patient feel supported and capable of managing the small, mundane aspects of her care. This shows how sense of agency is shaped in relation to trust and support from healthcare professionals. However, the patient’s sense of control was also relationally influenced by the healthcare system and socio-political forces (e.g., COVID-19 and the threat of a strike). The connection between personal health needs and broader social disruptions indicates that sense of agency and control are dependent on external factors.

The organized interaction between the patient and her healthcare team enhanced her feeling of security and confidence in the treatment process. Professionalism and attention to detail are evidenced by clear communication, which can help reduce anxiety and foster an experience of collaboration between patient and care providers. Our findings resonate with those of previous quantitative studies showing that patients who perceive that they are provided information based on their needs feel less anxious and have more control over treatment decisions (Shabason et al., 2014).

### Episode 4: Taking Control With Data—A Turnaround

The fourth episode demonstrates how an oncologist and a fellow patient with an education in healthcare contributed to Satu’s decision to read her own medical records.

My attitude and conduct in terms of searching for and utilizing information relating to my illness changed during the first two months after the diagnosis. At first, despite my natural interest in adopting and utilizing new knowledge, I did not read my test results on MyKanta, the national electronic health records platform used by both patients and health care professionals in Finland. I felt worried, unsure and incompetent, and did not want to read something I would not understand. This was also the advice of a doctor with whom I had spoken during the early examinations. When I met the oncologist five weeks after the first diagnosis, she encouraged me to read MyKanta. She explained that the results would not appear there if their quality was such that it would be better to hear them from the doctor. Also, Eeva encouraged me to read MyKanta as she had done along the way, from her early diagnosis on.

When the chemoradiation therapy started and I felt I had a better grip on many issues, I began to follow my medical records, such as my blood test results, very closely. I discussed them proactively with the doctors and nurses. When I was leaving the hospital after my second surgery, the doctor considered the length of time needed for home medication to prevent blood clots. I proposed that it would not be necessary to write a new prescription, because I had a couple of doses of medicine left from my previous surgery. I also planned to continue exercising immediately. The doctor agreed. This made me feel heard and valued.

This episode shows how, initially, Satu felt worried, unsure, and incompetent and chose not to engage with her medical records. After encouragement from the oncologist and Eeva, Satu began proactively following her medical records and test results. Kayastha et al. (2018) found that reading electronic medical records empowers cancer patients receiving treatments to ask clinicians more questions. In this episode, Satu’s active engagement, along with discussing her findings with her doctors and nurses, gave her a renewed sense of agency and control, enabling her to feel more in charge of her care and better informed about the progress of her treatment. Kayastha et al. (2018) found that reading medical records online relieves patients’ anxiety and increases their comprehension of their health situations.

Our analysis of this episode shows how the patient’s initial reluctance to read her medical records reflects the broader cultural tendency to rely on medical professionals for guidance, especially when facing overwhelming health concerns (Blumenthal-Barby, 2017). The oncologist’s encouragement to engage with the MyKanta service indicates a cultural shift toward patient empowerment, with patients having the tools and support needed to take ownership of their health. Healthcare systems increasingly encourage patients to be active in their own care, which reduces the hierarchy between doctors and patients (see Grünloh et al., 2018).

According to our interpretation, Satu’s evolving sense of control was influenced by her relationships with a professional and a peer, highlighting the relationality of agency. A peer who had gone through a similar experience (Eeva) provided support and practical advice, and the oncologist’s guidance fostered trust and reinforced self-management. Satu’s interaction with the doctor, in which her suggestion regarding home medication was accepted, further reinforced the importance of collaboration, showing that control and agency can be strengthened through positive, responsive relationships with professionals and peers. Lacking the aspect of patients’ interaction with healthcare professionals, Delbanco et al. (2012) showed, in a quasi-experimental study setting, how providing cancer patients with electronic access to their doctors’ notes was connected to an increased sense of control over their care.

We conclude that open and supportive communication between patients and healthcare professionals plays a critical role in the development of a patient’s sense of agency and control and in building trust in the process of care.

### Episode 5: Negotiating Control in a Rectosigmoidoscopic Examination

The fifth episode illustrates how Satu’s negotiation with the nurse regarding pain medication during the endoscopy generated a sense of control for her in a clinical setting.

One year after my major surgery, the cancer control examinations included a rectosigmoidoscopic examination. My previous experience with the procedure made without anaesthesia had not been at all good. I asked the nurse to give me strong pain medication. The nurse said that, based on his experience, he was not sure if strong medication would be needed in my case. He proposed that he would have everything ready for giving it to me but would only do so if I said that I needed it. I agreed with his suggestion. The surgeon, whom I highly trusted and appreciated based on my previous experiences, joined us after this negotiation.

During the examination, we chatted about a variety of topics, such as cross-country skiing. The surgeon showed me how artificial intelligence (AI) is utilized in the examination, which I found extremely interesting. No medication was needed. I felt good about the experience.

In this episode, Satu experienced a strong sense of agency by requesting pain medication based on a previous negative experience. The negotiation with the nurse, where the decision to administer medication was left to Satu, reinforced her sense of control.

According to our collective analysis, this episode reflects a cultural emphasis on patient-oriented care, in which patient preferences and comfort are prioritized (see Bolster & Manias, 2010). The nurse’s approach to keeping the medication on standby while respecting the patient’s decision highlights a collaborative healthcare culture (Schroeder et al., 2022). The surgeon’s demonstration of AI during the procedure reflects how digitalization and technological integration in healthcare are becoming more commonplace, aligning with broader societal acceptance of technology in medicine.

In our view, the patient’s sense of control is relationally constructed through interactions with the nurse and surgeon. Negotiation with the nurse emphasizes shared decision making, allowing the patient to maintain agency. The trust built with the surgeon from previous positive experiences enhances the patient’s comfort, showing that agency is relational and rooted in trust and open communication.

The negotiation with the nurse also exemplifies collaborative communication, as the patient’s preferences were respected despite the nurse offering a different perspective. Saltbæk et al. (2019) also found that constructive disagreements between patients and nurses enhance collaborative relationships between patients and healthcare professionals. The surgeon fostered an experience of ease by engaging in non-medical conversation and discussing the use of AI, creating a personal connection with the patient during the procedure. This indicates how personalized care and informative communication can improve a patient’s experience and increase their sense of control.

## Discussion and Conclusions

Our study shows how interaction with healthcare professionals, as well as lack thereof, shapes the process of losing and regaining a sense of control. This finding emphasizes the importance of human interaction regarding patients’ sense of control. Our detailed analysis showed how technology-driven and unidirectional communication and the subsequent lack of human interaction with healthcare professionals led to a cancer patient first losing her sense of agency and control upon receiving the diagnosis. This contrasts with the cultural shift toward self-sufficiency in understanding patients as agentic actors (From, 2015). Our detailed analysis depicted how the patient gradually regained her sense of agency and control over a long period of time as a result of dialogic, respectful, and participatory interactions with healthcare professionals, including nurses, doctors, and a peer patient. This allowed the patient to understand herself as an agentic and purposeful actor (Acke & Meganck, 2022; Deans, 2019), which was connected to better well-being and health outcomes (Street et al., 2009).

Our analysis illustrates how the process of regaining sense of agency and control was first shaped by the supportive and informative relationship with the nurse, who provided opportunities to ask questions and be involved in decision making. This practice reflects a more patient-oriented approach to care that is culturally valued in modern healthcare systems (Castro et al., 2016). Secondly, the patient’s sense of control was strengthened by another healthcare professional, the oncologist responsible for the overall treatment, who clarified the different roles and responsibilities involved in the cancer treatments. This is illustrative of patient-centered care, which is designed to address both the emotional and practical needs of patients (Mohammed et al., 2016). Later, regaining a sense of control was reinforced by a peer patient who encouraged the patient to follow her medical records and test results, which empowered her to discuss these with doctors and nurses. Finally, regaining a sense of control was strengthened through negotiating pain medication use with a nurse, as the patient’s preferences were respected, which is illustrative of a collaborative healthcare culture (Schroeder et al., 2022).

In prior empirical research, sense of control has often been conceptualized as a static phenomenon (Dickerson et al., 2011; Gibbons & Groarke, 2018; Henselmans et al., 2010; Kayastha et al., 2018; Monforte-Royo et al., 2018; Mystakidou et al., 2015). Our study challenges this view by showing how sense of control is dynamic and shaped in and through interactions within the healthcare system. Our study contributes to the illness management literature on cancer patients’ experiences (Greenhalgh, 2017; Gross, 2012; Horlick-Jones, 2011; Riessman, 2015; Wanat et al., 2016) from a social constructionist, contextual, and relational perspective. Whereas previous literature has theorized the concepts of sense of control and sense of agency as closely connected (Chambon & Haggard, 2012; Gallagher, 2007; Pacherie, 2007) and co-constitutive (Stapleton, 2018), our study contributes to both literatures by illustrating how patients’ sense of control is shaped by agency positions (Schlieper, 2022) that healthcare professionals either offer to patients, negotiate with them, or sideline altogether. Overall, we demonstrated how healthcare professionals’ resources and their capacity to engage in interaction with patients served as a precursor of and constitutive for patients’ sense of control over their life, health, and illness.

Our findings also add new insights into how the COVID-19 pandemic shaped patients’ sense of agency. Previous research has shown that delays in diagnosis and treatment occurred during the pandemic (Riera et al., 2021). Our findings suggest that the pandemic provided a new framework for patients to perform agency and feel a sense of control over another potentially fatal condition where pandemic-related procedures felt more tangible (such as wearing a mask) compared to their possibilities to control the progression of their cancer.

The findings of our study support the argument that healthcare systems need to move beyond merely delivering medical treatment by actively supporting patients’ perceptions of themselves as agentic actors who can manage their life, health, and illness. Healthcare professionals are powerful actors, whose engagement with patients is not just about offering good medical care and giving proper and timely information. The way in which the diagnosis and treatment processes are communicated in interaction with patients is fundamental for patients’ possibility to understand themselves as active, capable individuals (Campbell & Maynell, 2010), who perceive themselves as agentic citizens with rights and duties, despite having a serious illness (Beaver & Booth, 2007; Vogel et al., 2009).

Our research gives reason to pay attention to how healthcare systems that prioritize efficiency and automation through technology-driven solutions risk alienating patients, turning them into passive recipients of standardized procedures (Cruz, 2023). This detachment can erode trust, increase anxiety, and diminish patients’ ability to understand and advocate for themselves in their care and treatment (Bailo et al., 2019). Therefore, healthcare institutions must carefully strike a balance between efficiency and relational care (Farr & Cressey, 2015; Stewart et al., 2024). While streamlined processes are necessary in healthcare, they should be integrated with spheres that enable meaningful human interactions (Lenzen et al., 2018).

From a societal impact perspective, more research is needed to understand how patients diagnosed with cancer experience and construct their sense of agency and control. This knowledge could inform the development of tailored support systems and interventions that empower patients, improve their quality of life, and enhance their ability to navigate the challenges of their diagnosis and treatment in healthcare.
